# Darling, the doctor says I slept well but I still have headache in the morning: an actigraphic study in couples

**DOI:** 10.1186/1129-2377-14-S1-P145

**Published:** 2013-02-21

**Authors:** S Seidel, S Frantal, P Oberhofer, T Bauer, N Scheibel, F Albert, T Casjens, G Klösch, J Zeitlhofer, C Wöber

**Affiliations:** 1 Department of Neurology, Medical University of Vienna, Austria; 2 Department of Medical Statistics, Medical University of Vienna, Austria

## Introduction

Morning headache (MH) affects about 5 % of the general population [1] and have been related to insomnia symptoms. Snoring and obstructive sleep apnea syndrome (OSAS) are frequent sleep disturbances and may cause also impaired sleep quality in bed partners.

## Purpose/background/objectives

To date, prospective data on subjective and objective sleep quality in individuals with MH are lacking. Hence, the objective of this prospective actigraphic study was to compare sleep data of nights preceding days with and without MH in habitual snorers and their bed partners.

## Methods

We recruited habitual snorers and their non-snoring bed partners via newspaper articles. The participants completed a semistructured interview, filled in questionnaires about sleep quality, daytime sleepiness, depression and anxiety. Simultaneous actigraphy and sleep diaries were recorded during a 14-day period in these couples.

## Results

Forty five (11 female) snorers and 45 (34 female) bed partners with a mean age of 47±13 and 43±12 years were included in this study. Apnea screening yielded snoring without OSAS, mild OSAS, moderate OSAS and severe OSAS in 27 (60%), 8 (18%), 3 (7%) and 6 (15%) snorers. MH occurred on 6.3% and 4.9% of the recorded days in snorers and bed partners, respectively. In snorers, sleep efficiency (85±9 vs. 84±9, p=0.5) and fragmentation indices (34±16 vs. 36±14, p=0.5) did not differ significantly between nights followed by MH and nights not followed by MH. Bed partners showed a significantly higher sleep efficiency (86±8% vs. 89±6%, p=0.04) and lower fragmentation index (33±16 vs. 26±12, p=0.01) during nights, which were followed by MH compared to nights not followed by MH.

## Conclusion

In contrast to previous reports our prospective data do not confirm the relationship between insomnia and MH. In fact, bed partners of habitual snorers had even slept more efficiently if they reported MH the following day.
